# Supplementary material for: A new genomic framework to categorize pediatric acute myeloid leukemia
Source: Nat Genet. 2024 Jan 11;56(2):281–93. doi: 10.1038/s41588-023-01640-3 (PMC10864188; doi:10.1038/s41588-023-01640-3)
Supplement: Supplementary file 1 — Supplementary Figs. 1–3. [file 41588_2023_1640_MOESM1_ESM.pdf]

nature genetics

Article https://doi.org/10.1038/s41588-023-01640-3

A new genomic framework to categorize pediatric acute myeloid leukemia

In the format provided by the authors and unedited

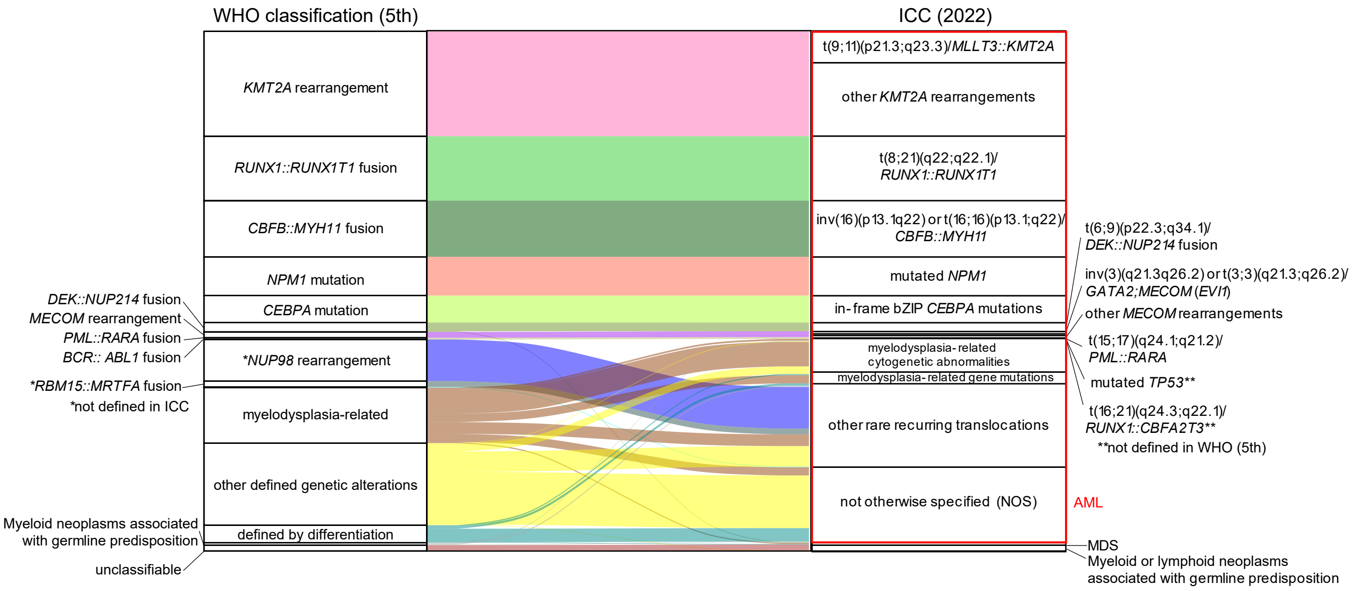

Supplementary Fig. 1 | Comparison between the WHO classification and International Consensus Classification (ICC).

Colors of the ribbon plot represent the WHO classification of samples in the pediatric AML cohort (n=887).

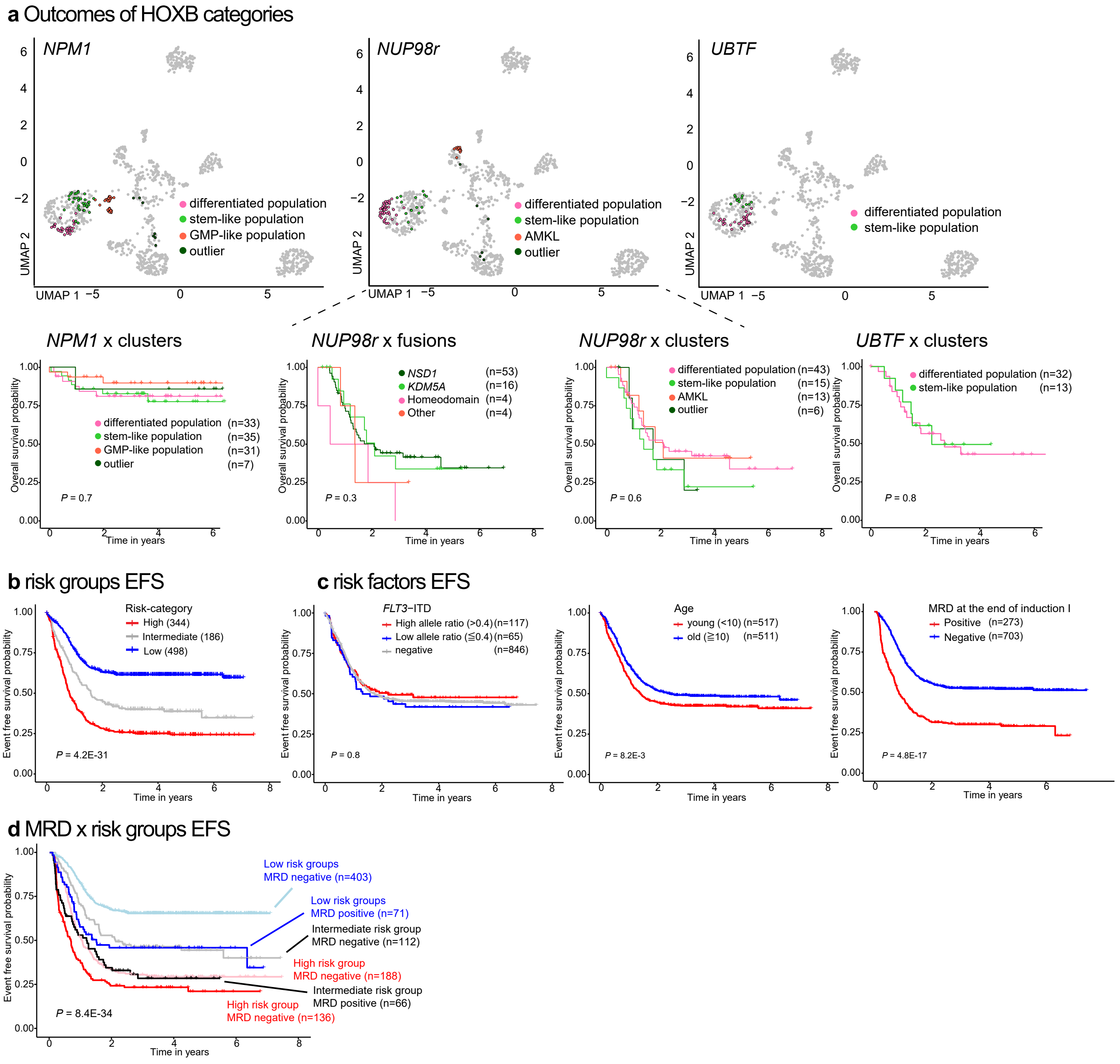

Supplementary Fig. 2 | Supplementary analysis of clinical outcome of the AAML1031 cohort

a. Distribution of *NPM1* (**left**), *NUP98*r (**middle**), and *UBTF* (**right**) on UMAP plots, with dot colors representing clusters the samples belong to (**top**), and Kaplan-Meier curves of overall survival according to transcriptional clusters or *NUP98*r fusion partners (**bottom**). **b**. Kaplan-Meier curves of event-free survival of patients in each risk group. **c**. Kaplan-Meier curves and statistical significance of event-free survival with known prognostic factors of *FLT3*-ITD (internal tandem duplication) status (left), age (middle), and MRD (measurable residual disease) positivity at the end of the induction I (right). **d**. Kaplan-Meier curves of event-free survival of patients in six risk strata using risk groups (Low-Intermediate-High) and MRD positivity. For all survival curves, statistical significance was assessed by the log-rank test, and *P* values are shown in the plots.

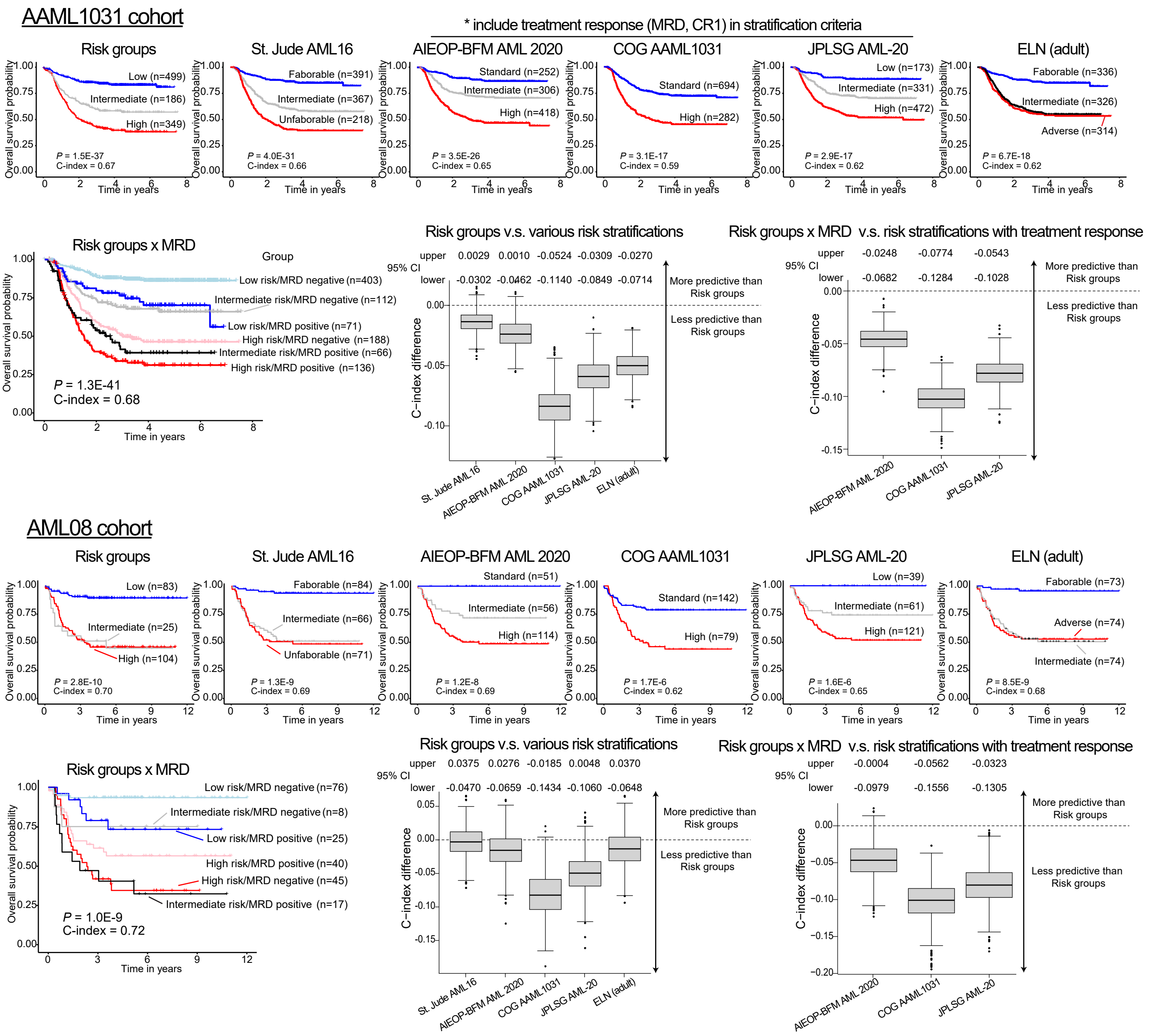

Supplementary Fig. 3 | Comparison of risk groups with various risk stratifications used in clinical trials

Assessment of risk-stratification strategies in the AAML1031 cohort (**top**) and the AML08 cohort (**bottom**) by bootstrapping (1,000 times). Bootstrap confidence intervals are computed as the 2.5 and 97.5 percentiles of the distribution of index differences between the proposed risk stratification and previously published risk stratifications. Lines of the box represent 25% quantile, median, and 75% quantile of the bootstrapping. The upper whisker represents the higher value of maxima or 1.5 x IQR, and the lower whisker represents the lower value of minima or 1.5 x IQR. Kaplan-Meier curves of overall survival of patients by referenced trial risk stratification and the comparison with risk groups in this study using bootstrapping (c-index scores) are shown. For clinical trials including treatment responses in the risk stratification (AEIOP-BFM, COG, JPLSG), the comparison with six strata (risk groups x MRD: measurable residual diseases) in this study is also shown. For all survival curves, statistical significance was assessed by the log-rank test, and *P* values are shown in the plots. For survival analysis involving MRD status, patients with available MRD status (MRD+:n=273, MRD-: n=703) are included.
