# Supplementary material for: A new genomic framework to categorize pediatric acute myeloid leukemia
Source: Nat Genet. 2024 Jan 11;56(2):281–93. doi: 10.1038/s41588-023-01640-3 (PMC10864188; doi:10.1038/s41588-023-01640-3)
Supplement: Supplementary file 2 — Reporting Summary [file 41588_2023_1640_MOESM2_ESM.pdf]

nature portfolio

Corresponding author(s): Jeffery M. Klco

Last updated by author(s): Nov 30, 2023

Reporting Summary

Nature Portfolio wishes to improve the reproducibility of the work that we publish. This form provides structure for consistency and transparency in reporting. For further information on Nature Portfolio policies, see our Editorial Policies and the Editorial Policy Checklist.

Statistics

For all statistical analyses, confirm that the following items are present in the figure legend, table legend, main text, or Methods section.

n/a	Confirmed	
☐	☒	The exact sample size (*n*) for each experimental group/condition, given as a discrete number and unit of measurement
☐	☒	A statement on whether measurements were taken from distinct samples or whether the same sample was measured repeatedly
☐	☒	The statistical test(s) used AND whether they are one- or two-sided<br>*Only common tests should be described solely by name; describe more complex techniques in the Methods section.*
☐	☒	A description of all covariates tested
☐	☒	A description of any assumptions or corrections, such as tests of normality and adjustment for multiple comparisons
☐	☒	A full description of the statistical parameters including central tendency (e.g. means) or other basic estimates (e.g. regression coefficient) AND variation (e.g. standard deviation) or associated estimates of uncertainty (e.g. confidence intervals)
☐	☒	For null hypothesis testing, the test statistic (e.g. *F*, *t*, *r*) with confidence intervals, effect sizes, degrees of freedom and *P* value noted<br>*Give P values as exact values whenever suitable.*
☒	☐	For Bayesian analysis, information on the choice of priors and Markov chain Monte Carlo settings
☒	☐	For hierarchical and complex designs, identification of the appropriate level for tests and full reporting of outcomes
☐	☒	Estimates of effect sizes (e.g. Cohen's *d*, Pearson's *r*), indicating how they were calculated

Our web collection on statistics for biologists contains articles on many of the points above.

Software and code

Policy information about availability of computer code

Data collection	HiSeq Control Software for Illumina HiSeq2000 and HiSeq2500 with most recent version at the time of sequencing were used to collect DNA and RNA sequencing data reported in this study.
Data analysis	StrongARM pipeline (not versioned), CICERO version.0.3.0, RNAseqCNV version 1.2.1, Bambino version1.07, RNAindel version3.0.4, VEP version95, PeCanPie (not versioned), BWA (WGS: v0.7.15-r1140 and v0.5.9-r26-dev; WES: v0.5.9-r26-dev and v0.5.9), Picard tools version 1.65, CREST version 1.0, CONSERTING (not versioned), Samtools version 1.16, VarScan2 version2.3.5, DNAcopy version 1.52.0, GRIN version 2, GISTIC version 2.0.23, gnomAD version 2.1.1, HTSeq version 0.11.2, Limma version 3.50.3, SVA version 3.42.0, Seurat version 4.1.0, destiny version 3.10.0, GSEA version 4.2.3, MSigDB gene sets c2.all version 7.5.1, WGCNA version 1.70-3, DAVID version 6.8, CIBERSORTx (not versioned), rpart version 4.1.19, survival 3.3.1, R version 4.0.2, pheatmap version 1.0.12, ggplot2 version 3.3.6, survminer version 0.4.9, iAdmix (not versioned)

For manuscripts utilizing custom algorithms or software that are central to the research but not yet described in published literature, software must be made available to editors and reviewers. We strongly encourage code deposition in a community repository (e.g. GitHub). See the Nature Portfolio guidelines for submitting code & software for further information.

Data

Policy information about availability of data

All manuscripts must include a data availability statement. This statement should provide the following information, where applicable:

- Accession codes, unique identifiers, or web links for publicly available datasets
- A description of any restrictions on data availability
- For clinical datasets or third party data, please ensure that the statement adheres to our policy

Genomic analyses in this study are based on the GENCODE GRCh37/hg19, and gnomAD version 2.1.1 was used for classification for germline and somatic mutations. The genomic data and expression data newly generated in this study (RNA-Seq: n=221, WGS: n=58, WES: n=7) have been deposited in the European Genome-Phenome Archive (EGA, RRID:SCR_004944), which is hosted by the European Bioinformatics Institute (EBI), under accession EGAS00001005760. Subsets of the new data (RNA-Seq: n=221, WGS: n=53, WES: n=5) have been also deposited to St. Jude Cloud under Pan-AML study (https://permalinks.stjude.cloud/panaml). Details are found in Supplementary Table 1.

For previously published RNA-Seq data (n=393), 266 are available either on EGA or St. Jude Cloud (1-9) or from the original publication (10). For the other 127 published cases (11), we downloaded the BAM files from EGA (EGAS00001004701). For previously published WGS data (n=198), 106 from the original publications (2, 3, 6-9) are available on either EGA or St. Jude Cloud, and the other 92 published BAM files (11) were downloaded from EGA (EGAS00001004701). For the previously published WES data (n=273), 153 with data from the original publications (1-9) are available either on St. Jude Cloud or EGA, and the BAM files for the other 120 published cases (11) were downloaded from EGA (EGAS00001004701).

We also downloaded data for publicly available but previously unpublished RNA-seq data (n=86) on St. Jude Cloud under the PCGP study (https://permalinks.stjude.cloud/permalinks/PCGP, n=8) and the RTCG study (https://platform.stjude.cloud/data/cohorts?dataset_accession=SJC-DS-1007, n=78). Similarly, we obtained unpublished WGS data (n=82: RTCG) and WES data (n=2: PCGP, n=99: RTCG study).

The data generated by the TARGET initiative (12,13) (n=187), including additional samples from the AAML1031 trial (14)(n=1034), are also available under accession phs000218 (TARGET-AML) and phs000465 (TARGET sub-study, data is available as a part of phs000218), managed by the NCI, and were obtained through GDC Portal managed by NCI under the TARGET-AML study (https://portal.gdc.cancer.gov/projects/TARGET-AML). Information about TARGET can be found at http://ocg.cancer.gov/programs/target. These sequencing data are available through controlled access as part of the NIH Genomic Data Sharing Policy (https://grants.nih.gov/grants/guide/notice-files/NOT-OD-14-124.html) and data access is restricted for academic use.

References
1. PMID: 30926971, 2. PMID: 34301788, 3. PMID: 30262806, 4. PMID: 29146900, 5. PMID: 25730765, 6. PMID: 27798625, 7. PMID: 35176137, 8. PMID: 28112737, 9. PMID: 23153540, 10. PMID: 31350825, 11. PMID: 34778799, 12. PMID: 29227476, 13. PMID: 3076086 14. PMID: 35349331

Human research participants

Policy information about studies involving human research participants and Sex and Gender in Research.

Reporting on sex and gender	Information on patient sex was based on biological features and collected along with other clinical data. Patient sex data was provided in supplemental table 1. We did not assume the impact of sex on the biological features or clinical outcomes of pediatric acute myeloid leukemia, and sex-focused analysis was not performed in this study.
Population characteristics	Patients had received a diagnosis of pediatric acute myeloid leukemia (AML) and the ages at diagnosis range from 0 to 23.5 (median 9.3). Of 881 patients with known sex, 418 patients (47.4%) were female and 463 patients were male.
Recruitment	Tumor samples from patients with acute myeloid leukemia from the St. Jude Children's Research Hospital tissue resource core facility were obtained with written informed consent.
Ethics oversight	St. Jude Children's Research Hospital institutional review board (IRB).

Note that full information on the approval of the study protocol must also be provided in the manuscript.

Field-specific reporting

Please select the one below that is the best fit for your research. If you are not sure, read the appropriate sections before making your selection.

☒ Life sciences ☐ Behavioural & social sciences ☐ Ecological, evolutionary & environmental sciences

For a reference copy of the document with all sections, see nature.com/documents/nr-reporting-summary-flat.pdf

Life sciences study design

All studies must disclose on these points even when the disclosure is negative.

Sample size	No sample size or power calculation was performed. The study cohort was determined by available patient samples with the diagnosis of AML

Sample size	and appropriate informed consent. We also included patients with available sequence data on public databases (St. Jude Cloud, EGA, and GDC data portal), to establish a large pediatric AML cohort of 887 patients fully characterized by sequence approaches.
Data exclusions	We excluded possibly duplicated samples estimated from pairwise genotype concordance comparison as well as low quality sequence data with possible tumor-normal contamination estimated from variant allele frequencies of somatic mutations as well as transcriptional analysis, which are not included in the final cohort of 887 patients. For patients with multiple time points at diagnosis or relapses, representative data points with good data quality with higher tumor purity estimation were included to establish a cohort with unique 887 patients.
Replication	For the genetic profiling of the study cohort, we performed the same analytical pipeline for an individual clinical study cohort of the AAML1031 study (n=1034), confirming the similar patterns of the overall molecular categories. For the clinical outcome data analysis, results from the AAML1031 study were validated using the AML08 study cohort (n=221, independent from the AAML1031 cohort, a part of this study cohort).
Randomization	No randomization of patients was performed in this study utilizing retrospective profiling of patients with available materials or sequence data. No analysis depending on patient background was performed in this study.
Blinding	No blinding was performed in the enrollment of patients or data collection of public data. Blinding in group allocation and in the following analyses were not possible as the grouping is based on the molecular characteristics of individual patients.

Reporting for specific materials, systems and methods

We require information from authors about some types of materials, experimental systems and methods used in many studies. Here, indicate whether each material, system or method listed is relevant to your study. If you are not sure if a list item applies to your research, read the appropriate section before selecting a response.

Materials & experimental systems

n/a	Involved in the study
☐	☒ Antibodies
☒	☐ Eukaryotic cell lines
☒	☐ Palaeontology and archaeology
☒	☐ Animals and other organisms
☐	☒ Clinical data
☒	☐ Dual use research of concern

Methods

n/a	Involved in the study
☒	☐ ChIP-seq
☐	☒ Flow cytometry
☒	☐ MRI-based neuroimaging

Antibodies

Antibodies used	For the purification of the tumor population from patient samples, CD45dimCD33dim~positive population was sorted using the following antibodies. CD34 gating was added depending on the positivity of each patient sample.<br>CD45 PerCP-Cyanine5.5 (eBioscience cat# 8045-9459-120) 1:20<br>CD33 APC (eBioscience cat# 17-0338-42) 1:20<br>CD34 PE (Beckman cat# IM1459U) 1:5<br>Links:<br>CD45 PerCP-Cyanine5.5 (discontinued): https://www.thermofisher.com/order/catalog/product/8045-9459-120<br>CD33 APC: https://www.thermofisher.com/antibody/product/CD33-Antibody-clone-WM-53-WM53-Monoclonal/17-0338-42<br>CD34 PE: https://www.beckman.com/reagents/coulter-flow-cytometry/antibodies-and-kits/single-color-antibodies/cd34/im1459u
Validation	These antibodies were validated for detecting human proteins by the manufacturers using human peripheral blood mononuclear cells (CD45 and CD33) or KG1A cells (CD34). For each experiment, gating for tumor population (CD45dim x CD33dim~positive x CD34 variable) was confirmed using isotype controls.

Clinical data

Policy information about clinical studies
All manuscripts should comply with the ICMJE guidelines for publication of clinical research and a completed CONSORT checklist must be included with all submissions.

Clinical trial registration	NCT00703820 (AML08) and NCT01371981 (the AAML1031 study); retrospective analysis only
Study protocol	https://clinicaltrials.gov/ct2/show/NCT01371981, https://clinicaltrials.gov/study/NCT00703820
Data collection	AML08 trial was open from 8/2008 to 3/2017. Clinical and outcome data was obtained for AAML1031 trial from GDC data portal in June 2022. The original clinical trial started June 20, 2011, and the primary completion was March 31, 2019.
Outcomes	AML08 data was obtained from study PI and co-author Dr. Jeffrey Rubnitz and from PMID 31246522. Publicly available data for AAML1031 was obtained from GDC data portal was analyzed retrospectively as described in the manuscript.

Flow Cytometry

Plots

Confirm that:

☒ The axis labels state the marker and fluorochrome used (e.g. CD4-FITC).

☒ The axis scales are clearly visible. Include numbers along axes only for bottom left plot of group (a 'group' is an analysis of identical markers).

☒ All plots are contour plots with outliers or pseudocolor plots.

☒ A numerical value for number of cells or percentage (with statistics) is provided.

Methodology

Sample preparation	For patients with less than 60% blasts, cryopreserved patient samples (bone marrow, peripheral blood) were thawed in IMDM media containing 20% FBS and subjected to flow-sorting for the tumor population before sequencing.
Instrument	Cell sorting was performed using a FACSAria III instrument (BD Biosciences)
Software	FACSDiva 9.0 software (BD Biosciences) was used for data collection and gating for sorting.
Cell population abundance	Enrichment of the tumor population was confirmed flow cytometric analysis of the post-sorting samples (generally > 90%).
Gating strategy	Live cells were first gated using FSC-A and DAPI (BD cat# 564907) gating, followed by singlet gating (SSC-W x FSC-A). The myeloid population was further gated as CD45 dim x FSC variable population. CD34 gating for the blast population was considered depending on the positivity of the tumor population in each patient.

☒ Tick this box to confirm that a figure exemplifying the gating strategy is provided in the Supplementary Information.
